# AI without representation is just inequity at scale: on the exportation of unrepresentative artificial intelligence models to the Global South

**DOI:** 10.3389/frai.2026.1894116

**Published:** 2026-07-29

**Authors:** César Abelardo Tinco Aliaga, Myles Joshua Toledo Tan, Vasco Gerardo Hinostroza Fuentes, Hezerul Abdul Karim, Nouar AlDahoul

**Affiliations:** 1 Department of Electrical and Mechatronics Engineering, University of Engineering and Technology, Lima, Peru; 2 Department of Electrical and Computer Engineering, Herbert Wertheim College of Engineering, University of Florida, Gainesville, FL, United States; 3 Department of Epidemiology, College of Public Health and Health Professions and College of Medicine, University of Florida, Gainesville, FL, United States; 4 Biology Program, College of Arts and Sciences, University of St. La Salle, Bacolod, Philippines; 5 Department of Natural Sciences, College of Arts and Sciences, University of St. La Salle, Bacolod, Philippines; 6 Department of Computer and Information Science and Engineering, Herbert Wertheim College of Engineering, University of Florida, Gainesville, FL, United States; 7 Centre for Image and Vision Computing, Centre of Excellence for Artificial Intelligence, Faculty of Artificial Intelligence and Engineering, Multimedia University, Cyberjaya, Malaysia; 8 Department of Computer Science, Division of Science, New York University Abu Dhabi, Abu Dhabi, United Arab Emirates

**Keywords:** AI governance, algorithmic fairness, artificial intelligence equity, dataset representation, Global South

## 1 Introduction

A common assumption in discussions of artificial intelligence (AI) is that increasing demographic representation in training data is sufficient to mitigate bias. Under this view, failures in facial recognition, clinical classification, and language generation are treated primarily as problems of coverage that can be resolved through more inclusive sampling. While intuitively appealing, this assumption becomes problematic when models trained and validated within one sociotechnical context are deployed into others whose realities were never meaningfully represented during development. This limitation is particularly evident in health-related AI, where outcomes are shaped not only by biological variables but also by lifestyle and environmental factors that interact dynamically over time, challenging purely data-centric notions of representation (Tan et al., 2025).

Emerging evidence suggests that the issue extends beyond coverage alone. Large-scale audits of biometric and healthcare datasets reveal widespread deficiencies in fairness, privacy, and regulatory compliance, underscoring structural weaknesses in how datasets are constructed and evaluated (Mittal et al., 2024). Even when models are optimized to satisfy formal definitions of algorithmic fairness, their real-world effects may remain inequitable. Simulation-based studies of AI-assisted healthcare systems demonstrate that achieving parity in model performance across groups does not necessarily translate into equitable outcomes, particularly when broader sociotechnical dynamics are taken into account (Stanley et al., 2025). More fundamentally, reducing fairness to measurable performance across predefined groups risks oversimplifying the structural and intersecting determinants that shape real-world outcomes, including the compounded effects of identity, environment, and access to care (Tan and Benos, 2025).

These limitations are not confined to data alone. Modern machine learning systems frequently rely on spurious correlations that enable high performance within specific datasets but fail under distributional shifts, a phenomenon widely described as the “Clever Hans” effect (Kauffmann et al., 2025). At a deeper level, model representations themselves often diverge from human conceptual organization, lacking the hierarchical and multi-scale structure that supports robust generalization (Muttenthaler et al., 2025). In parallel, studies of large language models across different sociocultural contexts continue to document persistent biases shaped by underlying data distributions and societal structures (Jiang et al., 2025), while work on dataset construction highlights enduring challenges in achieving globally representative, ethically sourced data (Xiang et al., 2025). These biases are further amplified when models treat populations as discrete or homogeneous groups, ignoring the intersectional nature of identity and the contextual factors that mediate how data is generated, interpreted, and applied (Tan and Benos, 2025).

Taken together, these findings suggest that representation in AI cannot be reduced to demographic inclusion alone. Instead, it must be understood across multiple interdependent dimensions: data (what is collected and how), context (the systems in which models are deployed, including environmental and lifestyle conditions), epistemology (how models internally structure knowledge), and infrastructure (the regulatory and operational frameworks governing their use). Failures along any one of these dimensions introduce risk; failures across all four, as often occurs when models developed in the Global North are exported to the Global South, can transform technical limitations into mechanisms of inequity at scale.

As used here, the Global South refers less to a fixed geographic region than to positions of relative disadvantage in the global distribution of technological authority, resources, and control. It is not interchangeable with low- and middle-income countries, resource-constrained settings, or settings of limited regulatory capacity, since these categories may overlap yet often diverge, and it is not internally uniform, since countries and institutions may differ in technical capacity, regulatory maturity, data infrastructure, and ties to multinational technology providers. The concern is, therefore, not that a model moves between regions, but that it may cross between sociotechnical environments of unequal authority.

This argument builds on a body of critical scholarship on AI and the Global South. Work on decolonial AI has shown how the design and deployment of systems may reproduce historical patterns of power when authority and value remain concentrated in high-income settings (Mohamed et al., 2020). Studies of algorithmic fairness in the Global South have further suggested that adjusting model outputs alone can act as window dressing when the technical, social, and physical distance between developers and affected communities is left intact, as analyses of deployments in India indicate (Sambasivan et al., 2021). Recent work reinforces this concern across regions and domains, with assessments of AI governance in Sub-Saharan Africa reporting limited local control and calling for data sovereignty (Ayana et al., 2024), studies of health AI treating colonial history as a sociostructural determinant that shapes fairness before a model is evaluated (Asiedu et al., 2024), and analyses of the political economy of AI locating inequity in ownership, infrastructure dependency, and labor rather than in model performance alone (Regilme, 2024). These perspectives tend to address data, fairness, participation, and governance through governance and process frameworks. The contribution of this article is to connect these stages within a single cumulative account, in which representation may fail at any point in the AI lifecycle and in which such failures can compound rather than remain isolated.

**Figure 1:**
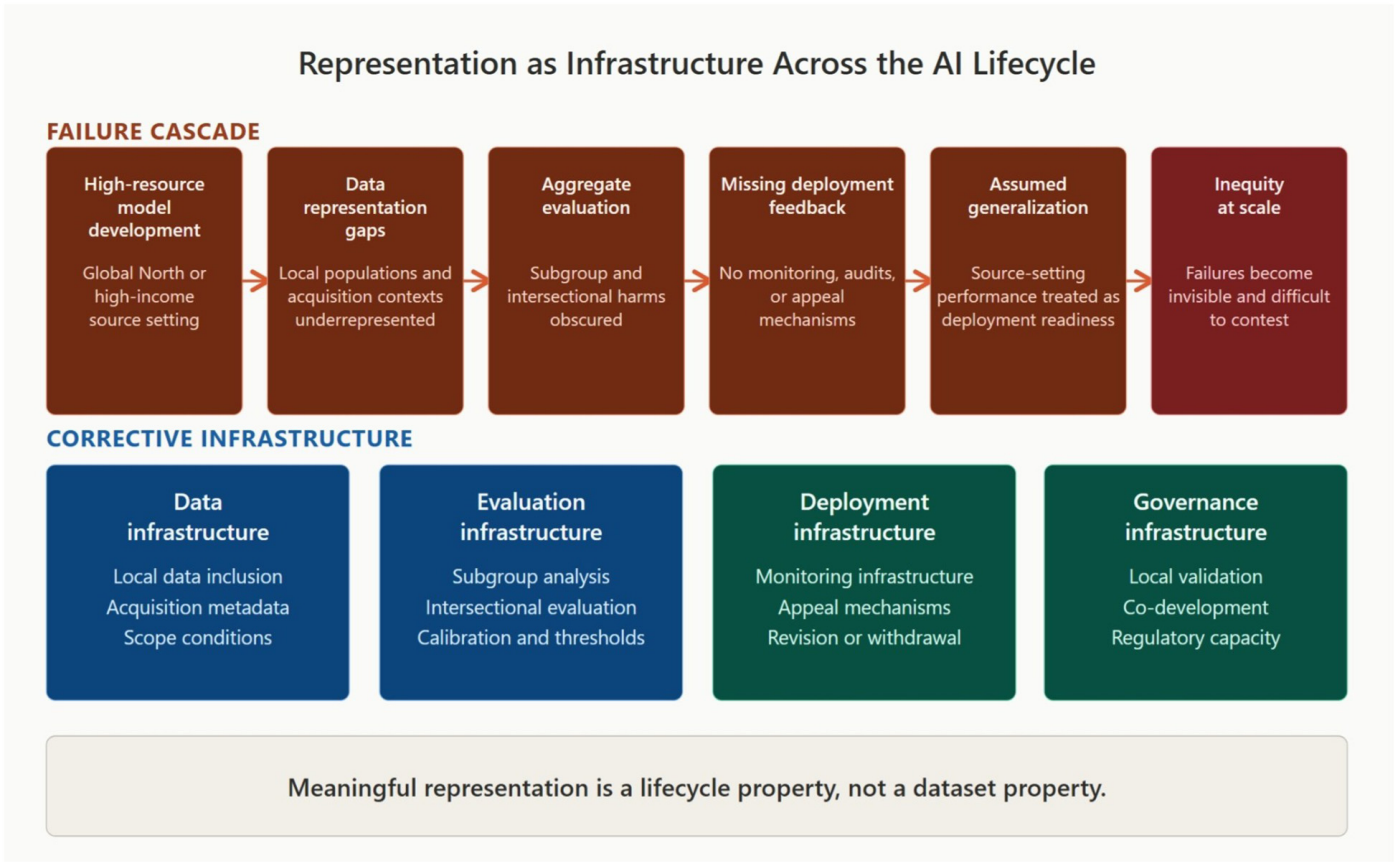
Representation as infrastructure across the AI lifecycle. The upper row illustrates a failure cascade in which AI models developed in high-resource settings move from incomplete data representation, aggregate evaluation, missing deployment feedback, and assumed generalization toward inequity at scale. Arrows indicate that these failures are cumulative rather than isolated. The brown boxes represent technical and institutional failures across development, evaluation, deployment, and generalization, while the red box represents the downstream consequence: inequity that becomes difficult to detect, contest, or correct. The lower row summarizes the corrective infrastructure needed across the lifecycle. Blue boxes represent data and evaluation infrastructure, including local data inclusion, acquisition metadata, scope conditions, subgroup analysis, intersectional evaluation, and calibration thresholds. Green boxes represent deployment and governance infrastructure, including monitoring, appeal mechanisms, revision or withdrawal, local validation, co-development, and regulatory capacity. The figure emphasizes that meaningful representation is not only a dataset property, but a lifecycle property.

This article argues that addressing inequity in AI requires an expanded notion of representation that integrates these dimensions. The sections that follow examine each in turn and contend that only a coordinated, systems-level response can prevent AI from reproducing, and amplifying, existing global asymmetries. Addressing these asymmetries therefore requires not only technical refinement but also transdisciplinary engagement across engineering, social science, and public health to ensure that AI systems reflect the complexity of the societies they are deployed in Tan and Benos (2025). The lifecycle framing of representation in this article, from data construction to evaluation, deployment feedback, generalization, and governance, is summarized in Figure 1.

## 2 Structural bias begins with data representation

Structural bias in AI systems often originates before model development, in the construction of datasets themselves. Representation at the data level is not limited to who is included, but extends to how data are captured, with what instruments, under which protocols, and what is excluded during preprocessing. These upstream decisions shape the statistical structure of the data and, consequently, the behavior of downstream models. Large-scale audits of biometric and healthcare datasets demonstrate that such design choices systematically affect fairness, privacy, and regulatory compliance, with widespread deficiencies observed across commonly used benchmarks (Mittal et al., 2024).

One important source of structural bias lies in the technical parameters of data acquisition. In medical imaging, AI models trained on chest radiographs have been shown to predict patient race with high accuracy, not through clinically meaningful features, but through correlations with acquisition and processing characteristics embedded in the images. These include factors such as exposure, contrast, and positioning, which reflect underlying disparities in clinical practice and data collection rather than biological signal (Lotter, 2024).

More broadly, machine learning systems are known to exploit low-level statistical regularities and shortcut features, reinforcing patterns that are incidental to the task but predictive within a given dataset. A similar phenomenon is observed in facial image analysis, where models can infer sensitive or unrelated personal attributes from non-demographic signals embedded in images, including camera artifacts, presentation choices, and contextual features. These findings highlight that datasets often contain far more information than intended, raising concerns about unintended information leakage and the difficulty of isolating meaningful signal from confounding factors (Tkachenko and Jedidi, 2023).

These acquisition-level biases are compounded by geographic and structural asymmetries in dataset curation. Dominant benchmarks in computer vision continue to exhibit limited global representation, with many datasets lacking diversity, relying on non-consensual data collection practices, and failing to capture environmental and contextual variability across populations (Xiang et al., 2025). Even when efforts are made to construct more balanced datasets, achieving meaningful representation requires deliberate, resource-intensive design, underscoring that diversity does not emerge automatically from scale.

In natural language processing, comparable patterns arise from the composition of large-scale pretraining corpora. These datasets are disproportionately derived from digitally active populations, often reflecting the linguistic patterns, cultural norms, and perspectives of urban, educated, and highly connected users. As a result, entire communities may be systematically underrepresented, leading to persistent biases and stereotypical outputs that mirror existing societal structures (Jiang et al., 2025).

These gaps are not confined to digital datasets. Even within clinical data collected in high-resource settings, substantial heterogeneity exists across institutions, with differences in patient populations, disease prevalence, and data collection practices producing non-identically distributed datasets. Such heterogeneity poses challenges for standard machine learning pipelines, which often assume stable and comparable data distributions across training and deployment environments (Shahid et al., 2026).

At a broader scale, limitations in physical and digital infrastructure directly constrain what data can be collected in the first place. In many regions, particularly across sub-Saharan Africa, existing datasets on poverty, health, and quality of life remain too coarse to capture local realities, reflecting a lack of high-resolution, context-specific data needed for actionable decision-making (Bettencourt and Marchio, 2025). In such settings, entire populations may remain effectively invisible within data-driven systems.

The downstream effects of these asymmetries are already observable. AI-based medical chatbots, for instance, have been shown to produce significantly different recommendations for identical clinical queries depending on geographic context, highlighting how data and deployment environments interact to produce uneven outcomes (Gumilar et al., 2024).

These structural deficiencies are unlikely to be resolved through increased data volume alone. On the contrary, recent work shows that indiscriminate incorporation of model-generated content into training data can lead to a degenerative process in which models progressively lose information about the underlying distribution, particularly at its extremes (Shumailov et al., 2024). Because underrepresented populations are already located in these distributional tails, they are disproportionately vulnerable to being erased in future training cycles.

Even under ideal conditions of dataset expansion, a further challenge remains: the metrics used to evaluate model performance may fail to capture the disparities that matter most. This limitation shifts the problem from data representation to evaluation itself, as the next section examines.

## 3 Evaluation metrics mask inequity

Standard evaluation metrics in machine learning often compress model performance into a single aggregate value, obscuring the possibility that a system performs well for some populations while systematically failing others. In medical imaging, this problem is particularly consequential. Chest radiograph classifiers have been shown to consistently and selectively underdiagnose underserved patient populations, with even larger underdiagnosis rates in intersectional subgroups defined by overlapping attributes such as race, sex, age, and socioeconomic status (Seyyed-Kalantari et al., 2021). Such findings illustrate a central weakness of aggregate evaluation: a model can appear clinically promising at the population level while producing patterns of error that would delay care for the very groups already at greater risk of medical neglect.

High aggregate scores can therefore function as a form of statistical concealment, masking clinically meaningful disparities behind averages that appear reassuring. Metrics such as AUC are useful for summarizing discrimination across thresholds, but they do not, by themselves, reveal whether a clinically chosen operating point produces equitable false-negative or false-positive rates across groups. This distinction matters because clinical decisions are not made across all possible thresholds simultaneously; they are made at specific thresholds that determine who receives follow-up, treatment, or reassurance. These disparities may be especially severe for intersectional subgroups, where overlapping dimensions such as race, sex, age, and socioeconomic status can compound vulnerability and make group-level averages even less informative (Seyyed-Kalantari et al., 2021; Tan and Benos, 2025; Xiang et al., 2025).

More broadly, formal fairness metrics can also mislead when treated as substitutes for context-specific judgments about welfare, access, and harm. Corbett-Davies et al. (2023) argue that prominent families of fairness definitions can produce decision policies that are detached from the policy goals they are meant to serve and may even harm the groups they were designed to protect.

The problem becomes deeper when strong benchmark performance is achieved not by learning the intended clinical signal, but by exploiting spurious correlations in the data. In radiographic COVID-19 detection, models were found to rely on confounding factors related to image acquisition and dataset source rather than medical pathology. These systems could appear accurate yet fail under evaluation in new hospital systems or rely on shortcuts that performance evaluation alone may not reveal (DeGrave et al., 2021). This is not an isolated pathology of COVID-19 datasets. More generally, standard performance metrics can be oblivious to whether a model has learned a valid problem-solving strategy or merely a Clever Hans shortcut that happens to work under the conditions of the benchmark (Lapuschkin et al., 2019).

Fairness constraints do not automatically solve this problem. In medical imaging, models can encode demographic attributes such as age, race, sex, and their intersections, and stronger demographic encoding has been associated with subgroup disparities in false-positive and false-negative rates. Methods that reduce demographic shortcuts can improve in-distribution fairness and produce models that appear locally optimal within the development dataset. However, Yang et al. (2024c) show that this local optimality often fails to hold in new test environments, where models selected for in-distribution fairness may not remain fair out of distribution. Thus, fairness measured during development may be less a property of the model itself than a property of the model-environment pair.

This problem of transferability is central to clinical deployment. Schrouff et al. (2022) show that fairness properties established in a source setting may not persist under distribution shift, especially when shifts are compound and involve simultaneous changes in demographics, labels, covariates, and clinical context. In one dermatology validation involving a teledermatology clinic in Colombia, subgroup performance gaps that were relatively small in the source setting became substantially larger in the target setting. Such findings are especially relevant to the Global South, where models imported from high-resource settings may encounter different disease prevalence, imaging practices, referral patterns, infrastructure constraints, and population structures.

Finally, the conceptual framing of evaluation itself may be poorly suited to clinical reality. Defining fairness as statistical equality across groups is not always clinically appropriate, because some group differences may reflect legitimate clinical variation rather than bias. Liu et al. (2023), therefore, argue that clinical AI fairness should be oriented toward equity rather than simple equality, with sensitive variables, fairness metrics, and mitigation strategies justified by clinical context and ethical reasoning rather than adopted as generic technical defaults.

Taken together, these findings show that the evaluation layer of AI development can reproduce and amplify the structural gaps already present in data construction. Without mechanisms to detect shortcuts, evaluate subgroup and intersectional performance, verify transferability, assess calibration and operating thresholds, and align metrics with clinical and social realities, models may systematically fail the populations they are designed to serve while appearing successful by standard measures. This points to a deeper structural absence: the feedback loops that would allow deployed models to be corrected are often missing, as the following section examines.

## 4 The missing iteration loop: AI without deployment feedback

The structural biases documented in data construction and the evaluation failures described in the previous section share a common condition: they persist in part because the systems that encode them are rarely corrected after deployment. Once an AI model is released into a financial platform, a hiring pipeline, or a public administration process, the feedback mechanisms that would allow errors to be detected and addressed are largely absent. Current methods lack strategies to address post-deployment scenarios of AI systems, and while there is significant research on trustworthy AI, there is only limited work on what happens after AI systems are deployed (Balendran et al., 2024). A systematic review of frameworks for AI procurement and monitoring found that while 84% of frameworks addressed planning phases, only 24% addressed continuous improvement, and none discussed when or how AI tools should be decommissioned (Khan et al., 2024).

This gap reflects a structural tendency in how machine learning systems are built. ML engineers push straight through to deployment, ignoring important productization and systems integration factors, and monitoring and feedback cycles that are important for continuous reliability and improvement over the product lifetime remain largely voluntary and inconsistently implemented (Lavin et al., 2022). The consequences can remain invisible until a critical failure occurs: in computer vision applications for automated recycling, a slight degradation in the initial model may not heavily influence early outputs, but when an uncommon input image appears, the app fails altogether (Lavin et al., 2022). The same dynamic explains why the Netflix Prize solution was never fully deployed despite its benchmark performance: real-world engineering costs and usage requirements were not anticipated during development.

In language model deployment, scaled-up and shaped-up models do not secure areas of low difficulty in which either the model does not err or human supervision can spot the errors (Zhou et al., 2024). Failures have been documented across domains including arithmetic, geographic knowledge, and scientific reasoning, where early models failed at simple additions and user-driven reliability is then seriously damaged because the model fails when the user thinks these digits were in the operating range (Zhou et al., 2024). As models become more capable and their outputs more fluent, the errors they produce become harder to detect without systematic feedback mechanisms.

This concern is particularly acute for large language models embedded in user-facing products. In such settings, the model is not merely a text generator but part of a sociotechnical interface that can allocate credibility, set boundaries around what information is surfaced or refused, and shape user reliance. Hinostroza Fuentes et al. (2026) describe this as an “institution-in-miniature”: a deployed LLM system may perform institutional functions such as gatekeeping, adjudication, and authority allocation, but without the corresponding safeguards of explanation, appeal, record keeping, or accountability. For exported AI systems, this matters because the harms of underrepresentation may be amplified not only through inaccurate outputs, but also through authoritative interfaces that make those outputs difficult to question, contest, or correct. Deployment infrastructure should therefore include provenance, explanation, appeal pathways, version awareness, and locally meaningful escalation mechanisms, especially when systems are used in high-stakes or institution-facing contexts.

In algorithmic hiring, many biases are difficult to fully understand because their techniques and methods are not easily visible, leaving many people unaware of why or how they are discriminated against, and the systems lack public accountability (Chen, 2023). The case of Amazon's ML-based hiring tool, which systematically discriminated against women and had to be withdrawn, illustrates how a system can operate at scale before its failures are recognized. Transparency would facilitate remediation when deviant algorithms are discovered, yet most hiring systems operate as black boxes with no structured mechanism for affected individuals to contest or correct outcomes (Chen, 2023).

Biometric identification shows the same pattern at national scale. In India, fingerprint-based authentication in the Aadhaar-enabled Public Distribution System has produced substantial authentication failures, which fall hardest on women, the elderly, and manual laborers whose fingerprints are worn by age or physical work, and which are compounded by poor connectivity and intermittent power in rural areas (Nagaraj and Prakash, 2022). The system assumed instant, one-size-fits-all verification, an assumption that does not hold where local infrastructure is limited and where a failed scan can place a household's right to food at risk.

In financial services, practical implementation requires continuous monitoring and adjustments, yet this remains challenging due to varying economic and social structures across developed and emerging economies (Vuković et al., 2025). In emerging markets, dominant platforms shape regulatory outcomes in ways that make traditional oversight difficult, and unchecked bias in AI systems can perpetuate inequalities in access to financial services, disproportionately affecting underserved populations (Vuković et al., 2025).

In public-sector algorithmic tools, the iteration gap carries direct distributional consequences. Environmental justice data tools have been shown to be highly sensitive to subjective model specifications, and state-run decision-making lacks accountability for affected communities, particularly when the state itself bears responsibility for perpetuating the injustices the tool was meant to address (Huynh et al., 2024). Most respondents underestimate the actual errors made by algorithms for credit scoring and recidivism prediction, and are willing to accept even fewer errors than they estimate to be occurring, suggesting that the absence of feedback is compounded by a systematic underestimation of the problem's scale (Rebitschek et al., 2021).

These dynamics are visible in Latin American welfare systems. In Colombia, the SISBEN targeting system uses an algorithm linked to dozens of public and private databases to score households for social programs, and it has been criticized for opaque profiling, undue administrative burdens on the poor, and the absence of clear mechanisms to appeal an adverse score (Garcia Alonso et al., 2022; Smart, 2024). A broader analysis of public algorithms across the region finds that automated welfare decisions often rely on proxies for economic status that generate indirect discrimination, and that the procedural ability to contest a decision is far weaker in the region than global fairness discourse assumes (Smart, 2024). Several of these systems, including childhood risk-scoring tools modeled on instruments from industrialized countries, were imported with little adaptation to local realities.

The iteration gap is also inseparable from questions of political economy and ownership. The export of AI involves vendors, intellectual property, cloud and compute infrastructure, licensing, procurement, and continued dependence on external technical support, and these arrangements determine where authority over a system actually resides. Analyses of the political economy of AI locate inequity in infrastructure dependency, labor, and the concentration of ownership in high-income settings rather than in model performance alone (Regilme, 2024). Assessments of AI governance in Sub-Saharan Africa similarly report that limited local data infrastructure can leave institutions dependent on foreign systems, with restricted authority to lead or contest how those systems are governed (Ayana et al., 2024). These conditions determine whether a deploying institution, rather than an external vendor, owns the imported model and the data generated locally during use, whether it has the means to inspect, modify, retrain, or withdraw the system, and who bears the recurring cost of local validation and monitoring.

One proposed structural response is to design correction mechanisms into the deployment architecture itself. By placing the human at the appeal stage, it becomes possible to allow for the correction of AI errors and the addition of relevant information that may be idiosyncratic, without requiring human involvement in every decision (Cohen et al., 2023). This appeal-based model mirrors legal oversight structures and offers a pathway for iterative correction without sacrificing efficiency. Its adoption remains limited, however, and its applicability in low-resource settings where regulatory capacity is weakest has not been established.

The iteration gap is most consequential in the Global South, where models imported from high-resource settings arrive without the contextual grounding, local feedback infrastructure, or regulatory oversight needed to detect when they are failing. The absence of a correction loop is not a neutral condition: it systematically advantages the populations and contexts represented in the original development environment, while leaving errors affecting other populations invisible and uncorrected. The result is not merely imperfect generalization but a structural amplification of existing inequities, a dynamic examined more fully in the following section.

These domains are not equivalent, and the argument does not assume they are. Clinical decision support, biometric identification, credit scoring, and public-sector welfare tools operate under different legal frameworks, different error structures, and different conditions of contestability. In clinical AI, error tends to be measured as biological harm or a missed diagnosis, whereas in welfare and identification systems the error is allocative, an exclusion from a benefit or a right, and the power asymmetry between an impoverished citizen and the state makes such decisions especially hard to contest. What these domains share is more fundamental than what separates them: a dependence on systems and design assumptions originating in high-resource settings, a tendency to treat social problems as technical faults correctable with more data, and a common failure of representation in which the conditions of the deployment context were never built into the system. It is this shared structure, rather than any single domain, that the lifecycle account is meant to capture.

## 5 Generalization failure as a symptom, not the problem

The failures documented in data construction, evaluation, and deployment feedback share a deeper structural origin: AI systems are often validated under conditions that are much narrower than the environments into which they are later introduced. Treating generalization failure as a technical defect to be corrected in isolation misses this point. Generalization is not a binary property that a model either has or lacks; it depends on the relationship between the development setting, the deployment setting, and the social or institutional task the model is meant to support. In healthcare, Futoma et al. (2020) warn against overly broad claims of generalizability and argue that the real question is whether a machine learning system is useful within the specific bedside context in which it is applied. The same principle applies more broadly: a model that works well in one institutional environment may fail when exported into another whose data, practices, infrastructure, and needs differ.

A central mechanism behind this failure is underspecification. Modern machine learning pipelines can return many distinct predictors that perform equivalently on held-out data, yet behave differently under stress tests or deployment shifts. D'Amour et al. (2022) demonstrate this problem across computer vision, medical imaging, natural language processing, electronic health records, and medical genomics, showing that models with similar i.i.d. validation performance can diverge substantially in robustness, fairness, and causal grounding. The implication is important: what appears to be a well-validated model may reflect the statistical convenience of a benchmark rather than a stable understanding of the task. In an underspecified pipeline, consequential differences in model behavior may be determined by seemingly minor choices such as random seed, initialization, or training procedure, rather than by principled alignment with deployment needs.

Shortcut learning compounds this problem. When training data contain hidden acquisition biases, models may learn these artifacts instead of the intended signal. Ong Ly et al. (2024) show across medical imaging, electrocardiograms, clinical notes, and lung auscultation data that model performance can be overestimated because models exploit data acquisition biases that do not generalize across settings. This is not merely a problem of weak model architecture. It is a problem of how passively collected data encode the routines, devices, protocols, and institutional practices of the sites that produced them. A model that learns these hidden features may appear accurate during internal validation but degrade when moved to a hospital, clinic, device ecosystem, or documentation practice that differs from the development environment.

The broader benchmarking ecosystem may reinforce these tendencies rather than correct them. Benchmarks do not merely measure progress; they steer it. Ott et al. (2022) show that many AI benchmarks quickly trend toward saturation, and that saturated benchmarks can become misleading measures of capability because further progress may reflect over-optimization to benchmark-specific characteristics rather than improvement that transfers to other data distributions. In natural language processing, Hupkes et al. (2023) similarly argue that high performance on traditional train-test splits does not imply robust generalization across domains, languages, tasks, shifts, or formulations of the same problem. These findings matter for representation because benchmarks define whose data count as evidence of progress. If benchmark ecosystems are narrow, saturated, or detached from the conditions of deployment, then models can appear to advance while remaining untested for the contexts where their failures are most consequential.

These dynamics become especially consequential when models developed in high-income settings are deployed in low- and middle-income settings. Yang et al. (2024b) evaluate the transfer of a United Kingdom-developed AI model to hospitals in Vietnam and show that models trained outside an LMIC context may struggle when local requirements, infrastructure, data availability, and clinical workflows differ from those of the development setting. Their work emphasizes that external generalizability is difficult because patient populations, healthcare disparities, local guidelines, data formats, and collection processes vary substantially across sites. In such cases, failure is not caused by the model alone. It reflects the assumption that a high-resource development environment can stand in for settings with different clinical realities.

Related work on HIC-LMIC bias mitigation reinforces this point. Yang et al. (2024a) show that collaborative models can produce divergent performance across HIC and LMIC settings, particularly when data imbalances are present, and that algorithmic bias mitigation can improve fairness while preserving diagnostic sensitivity. This is encouraging, but it also confirms the central concern: fairness and generalizability do not automatically travel with the model. They must be actively evaluated and adapted in the deployment context.

Postmarket distribution shift compounds this further. Koch et al. (2024) show that shifts in patient subgroups, image quality, ethnicity, sex, and comorbidity can affect the performance of medical AI systems after deployment, motivating the need for postmarket surveillance strategies capable of detecting clinically relevant distribution shifts. This matters especially for the Global South, where the technical and regulatory infrastructure needed for continuous monitoring may be limited. If models are imported faster than local surveillance capacity is built, then the populations least represented in the original development data may also become the least protected from silent model failure.

Taken together, these patterns suggest that generalization failure is not primarily a modeling problem but a structural one. It is rooted in how pipelines are specified, how benchmarks are constructed, whose contexts are treated as default, and whether deployment environments are given the authority and infrastructure to evaluate models on their own terms. Technical proposals such as selective deployment may reduce harm in some settings, but they also raise difficult questions about whether underrepresented groups may be excluded from beneficial systems because models were not built to serve them in the first place (Goetz et al., 2024). The deeper solution is not simply to withhold models from populations for whom they fail, but to restructure AI development so that those populations are represented in the data, evaluation, governance, and feedback systems that determine whether a model is ready for use. This is the challenge taken up in the following discussion. Figure 1 summarizes this progression from data-level gaps to evaluation failure, missing feedback, assumed generalization, and inequity at scale.

## 6 Toward representation as infrastructure: a discussion and call to action

The argument developed across the preceding sections converges on a single diagnosis: inequity in AI is not produced at any one stage of the pipeline, but through the compounding effects of all of them. Biased data, masked by aggregate metrics, left uncorrected by missing feedback loops, and assumed to generalize across contexts they never represented, produce harms that are structurally difficult to detect and institutionally easy to ignore. Demographic inclusion alone cannot interrupt this chain.

The central contribution of this article is the argument that representation must be reconceived as infrastructure, not a checklist. Data representation matters, but so does contextual representation: whether the social, institutional, and environmental conditions of a deployment setting were considered during development. Epistemological representation matters too: whether local knowledge systems, professional reasoning practices, and the expertise of affected communities were treated as legitimate inputs to model design. Infrastructural representation may matter most of all: whether the regulatory frameworks, monitoring systems, appeal mechanisms, and governance institutions needed to detect and correct AI failures exist in the places where models are deployed. A model that appears demographically balanced in development but arrives in a context with no post-deployment correction mechanism, no local validation process, and no accountability structure has not achieved meaningful representation. It has extended the reach of an unaccountable system. This lifecycle framing is summarized in Figure 1.

This distinction becomes especially consequential when AI systems cross borders. Models developed in high-income settings carry implicit assumptions about data infrastructure, institutional capacity, disease prevalence, documentation practices, and clinical workflows. When these models are exported, those assumptions travel with them. What does not always travel is the apparatus that made them correctable: the regulatory bodies, the feedback channels, and the professional communities with the authority and resources to contest model behavior and demand revision. The result is a structural asymmetry in which harm can accumulate silently, invisible to the developers who built the system and uncontestable by the populations who bear its consequences.

This asymmetry is also a matter of ownership and control. A model that the deploying institution cannot inspect, retrain, or withdraw, and whose locally generated data it does not own, has not been meaningfully represented in that setting, regardless of how balanced its training data may appear. Treating representation as infrastructure therefore requires that ownership, intellectual property, and institutional authority be addressed alongside data, evaluation, and monitoring.

Addressing this asymmetry requires that each stage of the pipeline be held to standards it currently lacks. Data documentation should extend beyond demographic composition to include acquisition conditions, institutional practices, and explicit statements of scope. Performance evaluation should disaggregate results by subgroup and intersectional category as a baseline requirement, with metrics selected in dialogue with affected communities rather than inherited uncritically from benchmark conventions. Deployment should be conditioned on the existence of monitoring infrastructure capable of detecting performance degradation over time, with defined thresholds for review, revision, and withdrawal. These are not optional ethical additions to AI development. They are the minimum conditions under which claims of responsible deployment become meaningful.

These standards can be made operational by specifying, for each layer of corrective infrastructure in Figure 1, the evidence that would be required before a system is considered ready for deployment. Data infrastructure could require documentation of collection settings, acquisition devices, and preprocessing decisions, together with an explicit statement of the population and context the data represent. Evaluation infrastructure could require intersectional subgroup analysis and local calibration at the operating threshold actually used in practice, rather than aggregate performance alone. Deployment infrastructure could require drift monitoring with defined review thresholds and accessible appeal mechanisms through which affected individuals can contest decisions. Governance infrastructure could require local regulatory review, documented institutional ownership of the model and of locally generated data, and the authority to modify or withdraw the system. Deployment readiness, on this view, is not a property of the model in isolation but a function of whether these conditions are met in the setting where it will operate, which allows representation as infrastructure to be assessed rather than merely asserted.

In this sense, representation as infrastructure should also include interface and organizational safeguards. For LLM-based systems in particular, deployment readiness should not be assessed only at the model layer, but also at the level of user interface, institutional workflow, procurement incentives, monitoring, incident response, and appeal mechanisms (Hinostroza Fuentes et al., 2026).

Appeal mechanisms warrant distinct consideration. The ability of individuals to contest algorithmic decisions that affect them is not merely a procedural courtesy; it is one of the primary channels through which localized failures surface and become correctable at scale. Without such pathways, errors that disproportionately affect underrepresented populations remain invisible to the systems designed to catch them. In contexts where broader regulatory infrastructure is limited, embedding appeal mechanisms directly into deployment architecture may represent one of the few structurally viable routes to accountability.

The Global South is not a passive recipient in this process. Researchers, clinicians, public health practitioners, engineers, and policymakers across low- and middle-income countries have developed deep expertise in managing complex systems under conditions of resource constraint, infrastructural variability, and high social need. This expertise is not incidental; it is precisely the kind of situated knowledge that AI development pipelines have systematically excluded, and precisely what is required to build models capable of functioning equitably across diverse deployment contexts. Participatory design approaches that position Global South institutions as co-developers rather than end users are therefore ethically preferable and epistemically necessary. Without the knowledge these institutions hold, models will continue to be validated against conditions that do not represent the environments in which they are most consequentially deployed.

Strengthening regulatory capacity in the Global South is equally urgent. The ability to evaluate, audit, and, if necessary, reject or modify imported AI systems requires institutional infrastructure that cannot be assumed to exist and cannot be built overnight. International partnerships and sustained investment in local AI governance capacity are, therefore, structural preconditions for equitable deployment, not optional supplements to it. Without them, the post-deployment monitoring and correction mechanisms argued for throughout this article will remain inaccessible to the settings that need them most.

Representation, understood as infrastructure, is ultimately both a technical and a political commitment. It requires that the communities most likely to be harmed by AI failures be included not only in training data, but also in the governance structures that determine how models are built, deployed, monitored, revised, and retired.

## 7 Conclusion

The failures this article has documented do not arise at isolated points in the development pipeline; they accumulate, and they conceal one another. Biased data collection is obscured by metrics that reward aggregate performance. Evaluation failures are masked by the absence of post-deployment feedback. The absence of correction mechanisms allows generalization failures to persist undetected in the very contexts where they cause the most harm. The result is not a series of discrete technical problems but a compounding structural one. As summarized in Figure 1, these failures accumulate across the AI lifecycle and require corrective infrastructure at each stage.

This gap is not distributed evenly. The communities least represented in AI development are also those most often denied the institutional resources needed to challenge the systems exported to them. Closing that gap requires more than better data. It requires that a model earn the right to deployment through accountability to the context it will serve, not merely through the demographics of the data on which was trained.

AI without representation is not merely incomplete. When exported into contexts it was not built to understand or serve, it becomes inequity at scale.
